# High Throughput Determination of Plant Height, Ground Cover, and Above-Ground Biomass in Wheat with LiDAR

**DOI:** 10.3389/fpls.2018.00237

**Published:** 2018-02-27

**Authors:** Jose A. Jimenez-Berni, David M. Deery, Pablo Rozas-Larraondo, Anthony (Tony) G. Condon, Greg J. Rebetzke, Richard A. James, William D. Bovill, Robert T. Furbank, Xavier R. R. Sirault

**Affiliations:** ^1^High Resolution Plant Phenomics Centre, Commonwealth Scientific and Industrial Research Organisation Agriculture and Food Agriculture and Food, Canberra, ACT, Australia; ^2^Commonwealth Scientific and Industrial Research Organisation Agriculture and Food, Canberra, ACT, Australia; ^3^ARC Centre of Excellence for Translational Photosynthesis, Australian National University, Canberra, ACT, Australia

**Keywords:** LiDAR, plant phenomics, above-ground biomass, NDVI, field experiments

## Abstract

Crop improvement efforts are targeting increased above-ground biomass and radiation-use efficiency as drivers for greater yield. Early ground cover and canopy height contribute to biomass production, but manual measurements of these traits, and in particular above-ground biomass, are slow and labor-intensive, more so when made at multiple developmental stages. These constraints limit the ability to capture these data in a temporal fashion, hampering insights that could be gained from multi-dimensional data. Here we demonstrate the capacity of Light Detection and Ranging (LiDAR), mounted on a lightweight, mobile, ground-based platform, for rapid multi-temporal and non-destructive estimation of canopy height, ground cover and above-ground biomass. Field validation of LiDAR measurements is presented. For canopy height, strong relationships with LiDAR (*r*^2^ of 0.99 and root mean square error of 0.017 m) were obtained. Ground cover was estimated from LiDAR using two methodologies: red reflectance image and canopy height. In contrast to NDVI, LiDAR was not affected by saturation at high ground cover, and the comparison of both LiDAR methodologies showed strong association (*r*^2^ = 0.92 and slope = 1.02) at ground cover above 0.8. For above-ground biomass, a dedicated field experiment was performed with destructive biomass sampled eight times across different developmental stages. Two methodologies are presented for the estimation of biomass from LiDAR: 3D voxel index (3DVI) and 3D profile index (3DPI). The parameters involved in the calculation of 3DVI and 3DPI were optimized for each sample event from tillering to maturity, as well as generalized for any developmental stage. Individual sample point predictions were strong while predictions across all eight sample events, provided the strongest association with biomass (*r*^2^ = 0.93 and *r*^2^ = 0.92) for 3DPI and 3DVI, respectively. Given these results, we believe that application of this system will provide new opportunities to deliver improved genotypes and agronomic interventions via more efficient and reliable phenotyping of these important traits in large experiments.

## Introduction

The rate of genetic gain per year for yield potential of wheat over the last two decades has stabilized at <1% per annum (Reynolds et al., 1999; Fischer et al., 2012). Various interventions have been proposed to maintain or improve this rate. Field phenomics, with its potential to non-destructively and remotely-sense crop traits associated with performance in a high-throughput fashion (White et al., 2012; Araus and Cairns, 2014; Deery et al., 2014, 2016; Rebetzke et al., 2016; Shakoor et al., 2017), has gained more attention as a promising intervention in recent years. Key physical parameters that are targets for field phenomics include canopy height, early ground cover, distribution, and maintenance of green leaf area, and biomass production.

The importance of canopy height and its relationship to harvest index (defined as the ratio between harvested grain and total above-ground biomass) are well known from the Green Revolution, where the introduction of dwarfing genes resulted in semi-dwarf wheat varieties with increased harvest index and yield (Reynolds and Borlaug, 2006). Canopy height is typically measured with a graduated stick or ruler by holding together a handful of stems from a representative part of a given experimental plot and recording the height to the tip of the spike for an average stem (ignoring the awns) (Rebetzke et al., 2013b). Plant height is elsewhere defined (perhaps more appropriately as canopy height) as the shortest distance between the upper boundary of the main photosynthetic tissues (excluding inflorescences) on a plant and the ground level (Pérez-Harguindeguy et al., 2016). Despite efforts to standardize the canopy height measurement, there is a larger component of subjectivity with operators potentially having a different perception of what constitutes the height of the canopy.

Canopy ground cover (GC) represents the fraction of the soil covered by the crop. High GC is necessary for intercepting light needed for growth, shading the soil to reduce soil evaporation (Fischer, 1981; Botwright et al., 2002; Richards and Rebetzke, 2002; Rebetzke et al., 2004; Mullan and Reynolds, 2010), and for weed competitiveness (Coleman et al., 2001). The GC assessments are generally made from early emergence until complete canopy cover. There are three standard methods for the estimation of GC: (1) Digital photographs taken at constant height over a representative area of the experimental plot and then processed with image analysis software for the classification of vegetation and non-vegetation pixels (Li et al., 2010; Mullan and Reynolds, 2010; Pask et al., 2012; Kipp et al., 2014); (2) Spectral indices such as the normalized difference vegetation index (NDVI), including active spectral sensors like the GreenSeeker® (Trimble, USA), which have shown strong associations with GC up to the stem elongation growth stage (Gitelson et al., 2002); and 3) Visual scores, where expert estimates of GC are made on ordinal scale (e.g., on a 1–9 scale, with 1 = no GC and 9 = complete GC).

Opportunities to increase the yield potential of wheat are now focusing on increasing above-ground biomass, while maintaining high harvest index (Reynolds et al., 2009, 2011, 2012). However, measurement of above-ground biomass requires cutting the culms at ground level for a defined portion of the experimental plot (normally 0.1–0.3 m^2^), and then weighing after drying in an oven until constant weight (Pask et al., 2012). The reliability and resulting confidence in these above-ground biomass measures are limited by: (1) the sample representing a small section of the experimental plot, which could be a misrepresentation if the plot is not uniform; (2) samples are destructive, thereby limiting the number of samples before destroying the entire plot; and 3) sampling and subsequent processing requires transport, drying, and manual handling, which contributes to sample loss and can be restrictive in large experiments. Thence, measurement of above-ground biomass is laborious and subject to large experimental error. Recent developments in remote and proximal sensing for high-throughput field phenotyping have led to proposed alternatives to destructive sampling, including the use of digital photography and NDVI sensors (Li et al., 2010; Pask et al., 2012), across multiple scales (Hawkesford and Lorence, 2017) using both aerial (see review by Yang et al., 2017) and ground platforms (Busemeyer et al., 2013; Andrade-Sanchez et al., 2014; Deery et al., 2014; Barker et al., 2016; Virlet et al., 2016; Kirchgessner et al., 2017; Liu et al., 2016).

Light Detection and Ranging (LiDAR) mounted on a field phenotyping buggy was recently proposed for quantifying a number of traits, including canopy height, GC and above-ground biomass (Deery et al., 2014). LiDAR, as an active sensor, confers many advantages over passive sensing (see also Lin, 2015), including: (1) operation regardless of ambient light conditions; and (2) direct measurement of canopy height and architecture. LiDAR-derived images can avoid some of the limitations of RGB images which have been used for these purposes, namely that changes in ambient light conditions and shadows can result in over or under exposure, thereby reducing image quality and data reliability (Deery et al., 2014).

While the use of LiDAR for the estimation of physical height and above-ground biomass in forestry applications is well established (Lefsky et al., 1999, 2002; Clark et al., 2004; Hyyppä et al., 2008; Lucas et al., 2008; Eitel et al., 2013; Kankare et al., 2013; Greaves et al., 2015), its application in crops is still in its infancy. Saeys et al. (2009) estimated crop density and spike number using statistical models for two LiDAR scanning frequencies. Another approach that has been widely adopted is the use of canopy height as a surrogate for crop biomass (Ehlert et al., 2008, 2009, 2010; Gebbers et al., 2011; Tilly et al., 2014). Most of these methodologies are based on the extraction of crop height using a surface differencing approach (Louise Loudermilk et al., 2009), where the digital terrain model is subtracted to the crop surface model. More recently, some authors have proposed the combined use of LiDAR-derived canopy height and reflectance information (Eitel et al., 2014; Geipel et al., 2014; Tilly et al., 2015). Also, the option of estimating canopy height using stereo reconstruction from aerial imagery (Bendig et al., 2014; Geipel et al., 2014; Aasen et al., 2015) and ground platforms (Salas Fernandez et al., 2017), has been proposed as an alternative to LiDAR. However, prediction of above-ground biomass from height is unlikely to be of benefit in breeding trials, where variation in plant height is commonly restricted. For this reason, alternative approaches are required to provide robust estimates of biomass production.

In this paper, we present the development and early application of the Phenomobile Lite™ (http://www.plantphenomics.org.au/services/phenomobile/) as an evolution of the original Phenomobile (Deery et al., 2014). The Phenomobile Lite, conceived as a manually-operated buggy, is designed to be lightweight, cost-effective, and transportable across multiple field sites, thereby providing reliable field phenotyping amenable to deployment in multi-site managed environment facilities for targeted trait and germplasm evaluation (Rebetzke et al., 2013a). We describe the algorithms developed for non-destructive measurement of canopy height, GC, and above-ground biomass using LiDAR data, and demonstrate the utility of the Phenomobile Lite and LiDAR for use in plot-scale phenotyping within genetics, physiology or agronomy studies, or in a plant breeding program.

## Materials and methods

### Phenomobile lite description and components

**Figure 1:**
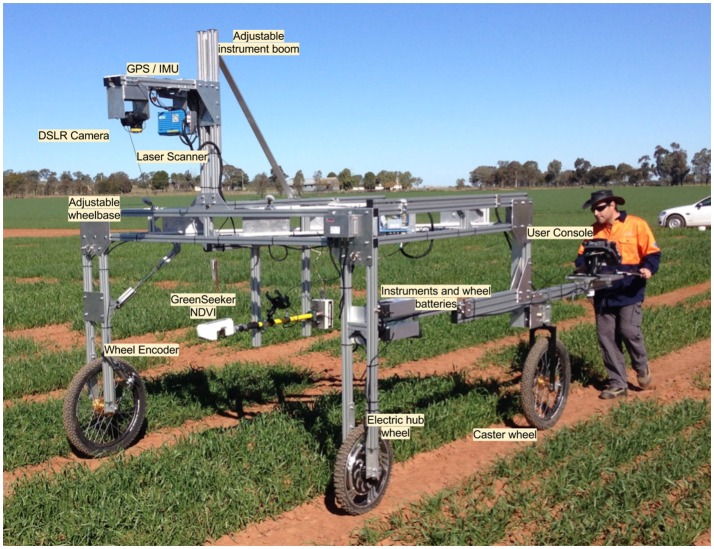
Phenomobile Lite comprising LiDAR laser scanner, digital single-lens reflex (DSLR) camera, GreenSeeker and touch-screen computer mounted on an aluminum frame with adjustable wheel spacing to accommodate different plot widths (1.75–2.20 m) and ground-clearance for canopy heights up to 1.5 m. The height adjustable sensor boom (2.0–2.5 m) enables data capture from crop emergence to maturity. The Phenomobile Lite is powered by an electric wheel and steered by an operator walking behind. Data is captured on a touch screen computer and processed through a web interface whereby the user processes the plot data in a semi-automated fashion (see Figures 2, 3).

The Phenomobile Lite is a portable buggy consisting of a lightweight extruded aluminum frame with three wheels and an instrument platform (Figure 1). The front-left leading wheel is powered by an electric motor with manual speed control and the rear wheel, trailing the front powered wheel, acts as a caster wheel for steering. The adjustable wheelbase on the Phenomobile Lite can accommodate different plot widths (1.75–2.20 m). The height-adjustable instrument boom (ground clearance of 1.5 m), located at the front of the frame, can be adjusted as the crop develops to maintain a constant distance above the canopy and restrict the plot within the field of view of the instruments. At the rear of the unit, the operator has access to a digital display with touchscreen for controlling the devices.

The Phenomobile Lite comprises the following instrumentation:

1. A high-frequency laser scanner or LiDAR. The model selected (SICK LMS 400-2000, SICK AG, Waldkirch, Germany) works on the phase-shift principle for estimating the distance. Light with a given wavelength that travels to an object and then back will be shifted in phase compared to the emitted light, being the phase-shift proportional to the distance between the sensor and the object. The laser operates at 650 nm (visible red light) and 4 mW of power, generating a spot diameter of *ca*. 2 mm at 3 m distance. The scanning rate is 270 Hz with an angular resolution 0.1°.
2. An inertial navigation unit (IMU) with GPS for registering the position and orientation of the LiDAR. The IMU (Spatial, Advanced Navigation, Australia) has 0.2° accuracy and 0.6 m horizontal accuracy when differential GPS corrections are provided. It can operate over a broad range of temperatures and presents a minimal form factor (37 grams).
3. Incremental wheel encoder (SICK DFV60A, SICK AG, Waldkirch, Germany) with a maximum angular resolution of 65,536 counts per revolution which translate to sub-millimeter linear resolution.
4. Computer with touch screen (Toughpad, Panasonic, Osaka, Japan).
5. Additionally, the Phenomobile Lite can integrate other instruments such an active NDVI sensor (GreenSeeker, Trimble, USA) and digital camera (Canon 6D, Canon Inc., Tokio, Japan), which are triggered by the control software based on traveled distance or time intervals.

The operating software and user-interface were developed in Java programming language (Oracle, https://www.oracle.com/java/index.html), designed for the non-technical user (i.e. intuitive and user-friendly). The experiments are typically organized in field layouts of columns and rows. Whereby, we considered columns to be experimental plots in the direction of the sowing and rows the position of each experimental plot within the columns. To acquire data, the user drives the Phenomobile Lite to the first plot of the experiment and sets the experiment name on the control software. Then, the user presses a start button and drives the Phenomobile Lite along the column, without any additional input. Maintaining a constant speed is not critical as the Phenomobile Lite registers the speed with the GPS/IMU and wheel encoder, which is later used to post process the data. A cruise control function is available to maintain the electric wheel at a constant speed. At the end of the column, the user presses “Stop” and turns the Phenomobile Lite to the start of the next column. A map is displayed in real time with the GPS track and an aerial display of the experiment. The direction of the operation is automatically taken into account based on the heading information from the IMU/GPS, which simplifies the data processing when the operation is done in zigzag mode. Once all the columns from the experiment are completed, the raw data is compressed and uploaded to the web server for processing.

### Phenomobile lite data capture and pre-processing

#### Data files

The data from the LiDAR and associated instruments are collected on the touch screen computer and stored in the following data formats:

1. LiDAR data is stored as binary files (one file per column). The data format was custom-made and is composed of a header and a collection of LiDAR scans with an associated header for each scan. The file header contains basic information about the location of the LiDAR unit and offsets with the GPS/IMU and other instruments. It also includes the configuration of the LiDAR device (scan rate and angular resolution). Then follows a sequence of scans, each one with a header defining information associated with each scan, such as the timestamp, encoder count, GPS/IMU position and orientation. Finally, the scan contains the range and intensity observations (generally 700 points per scan). With the LiDAR operating at rate of 270 scans per second, each file will contain a number of scans that will depend on the length of the column and speed of operation.
2. GPS/IMU position and orientation, containing the output from the GPS/IMU with GPS timestamps, is stored as comma separated values (CSV).
3. GreenSeeker NDVI measurements are linked to the GPS/IMU position and timestamps. These measures include the red and near-infrared reflectance as well as the derived vegetation indices calculated by the GreenSeeker unit (NDVI and RVI). This information is stored as CSV.
4. Coordinates for the trigger events for the RGB camera are stored as CSV. The images from the camera also contain GPS information such as approximate position and GPS timestamps used to link the trigger event with the actual image and therefore plot.

The files are identified by the name of the experiment, date, column/row coordinates and column number for unique identification. It is possible to add suffixes to denote multiple passes for a given column on a single day (e.g., column pass before and after biomass sample).

#### Processing workflow

**Figure 2:**
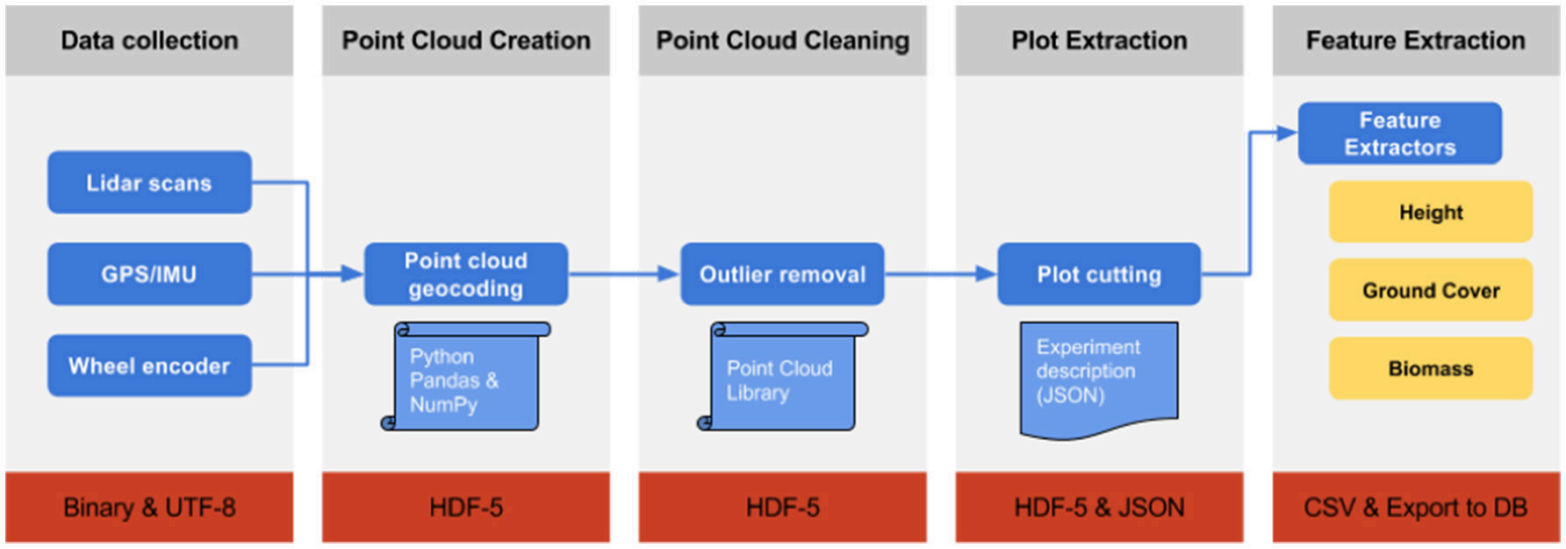
Schematic of the Phenomobile Lite LiDAR data processing workflow, whereby each box represents the following steps (from left to right). Data collection: Raw data collection with the Phenomobile Lite touch screen computer. Point Cloud Creation: the collected LiDAR data is converted into X,Y,Z coordinates. Point Cloud Cleaning: LiDAR data is cleaned and outlier points, resulting from partial returns. Plot Extraction: LiDAR data is segmented into experimental plots by the user, through a web interface (Figure 3). Feature Extraction: Trait data are extracted from the LiDAR data for each experimental plot. The outgoing data file format for each step is indicated in the respective red rectangle.

The processing workflow is designed as a pipeline with multiple steps (Figure 2) and was developed with Python 2.7 (Python Software Foundation, https://www.python.org/) and Go (Google, https://golang.org/) programming languages. The processing architecture is designed to be amenable to parallel computing and deployment into cloud infrastructures. The workflow is presented to the user as a web interface that provides access control, data upload, interactive visualization, plot selection and export of the results to standard formats. A web-based architecture allows the user to process the data without installing any specific software other than a web browser.

**Figure 3:**
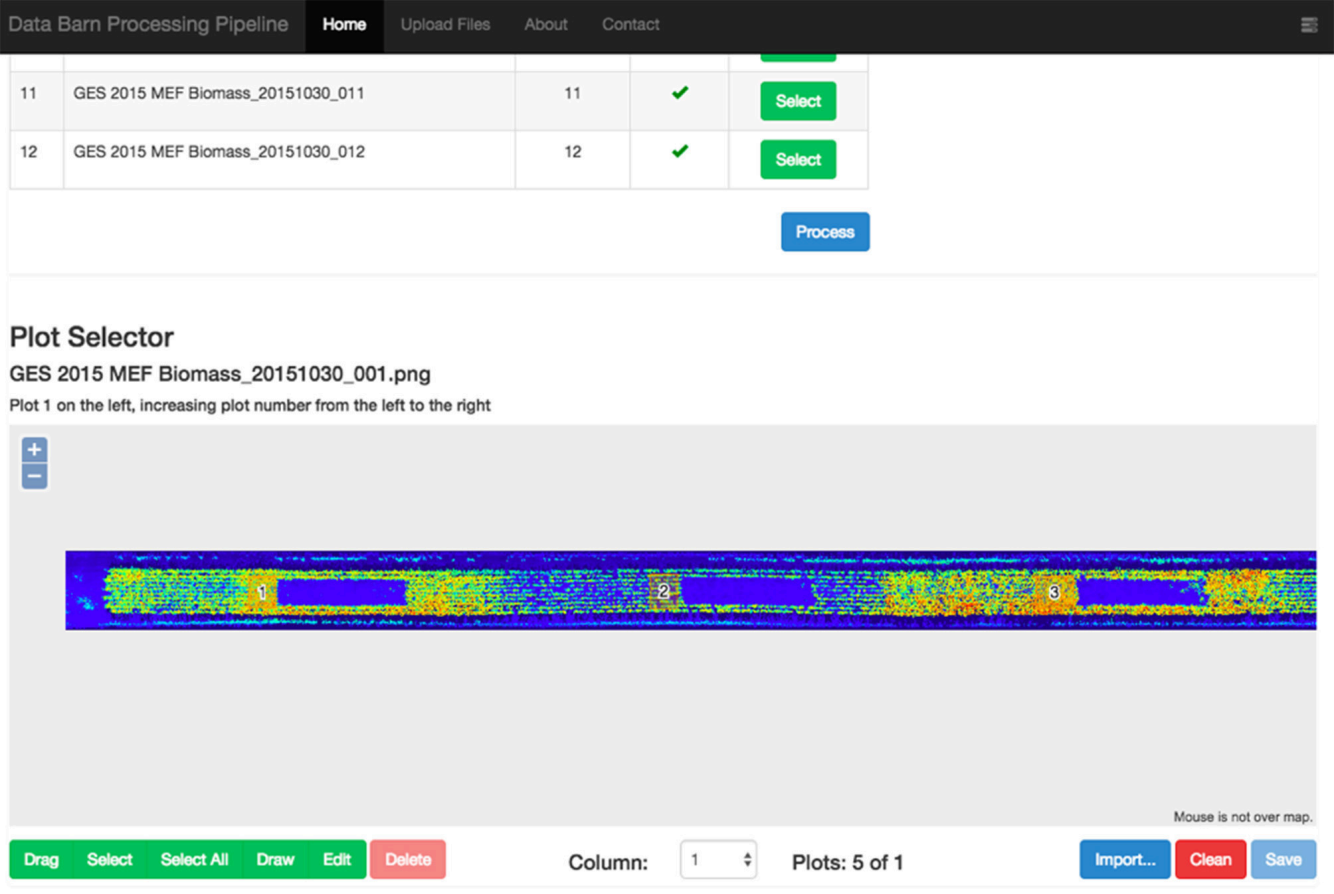
Web interface of the LiDAR data processing pipeline showing a rasterised point-cloud for a column of experimental plots. The user-selected sections of the experimental plots are denoted with numbered orange rectangles. Note how the user has avoided existing biomass samples (blue boxes to the right of the numbered orange rectangles).

The raw data, collected in the field and comprising the LiDAR scans, their location, and orientation from the GPS/IMU and wheel encoder, are compressed and uploaded to the web server. Once the data is uploaded, it is transformed from raw LiDAR returns (containing the range to the object and angle) into Cartesian coordinates, which creates the point cloud for the entire column with each point comprising an *x, y*, and *z* coordinate. Where: the *x* coordinate is the position across the column (along the width of the column); the *y* coordinate is the position down the column (along the length of the column); the *z* coordinate is the vertical position. The point cloud can contain a number of outliers and spurious points that usually appear when the laser hits the edge of leaves or in very bright light conditions. In the next step, the point cloud is filtered using Point Cloud Library (Rusu and Cousins, 2011) and the statistical outlier removal filter (Rusu et al., 2008). Once the point-cloud is clean, a rasterised version of the point-cloud is displayed on the web interface, thereby enabling the user to visualize all the experimental plots for a particular column (Figure 3). The user then draws a rectangle around every experimental plot, which defines the areas of interest for each plot on which the feature extraction algorithms will be applied. The user-supervised selection of the experimental plots enables the user to avoid areas of the plot that would normally be discarded in the field experiment (e.g., plot borders) and prevents measurements on areas where biomass has been removed for sampling or where a plot has been damaged (e.g., wheel tracks). The plot-selection could be automated in the future with image analysis techniques.

Once the areas delimiting the plots have been defined, the point-cloud representing each experimental plot is extracted. Finally, the point clouds associated with each plot are processed using different algorithms for the extraction of biologically meaningful measures representing traits of interest. Once the processes are completed, the pipeline generates a table with the associated measurements for each plot. The user can download these measures, linked to the traits of interest, as a CSV file or upload them to an existing online database or virtual laboratory such as SensorDB (Salehi et al., 2015).

The workflow, described in Figure 3, is controlled by a coordinator server that monitors the queue of the different tasks associated with the pipeline and delegates the tasks to different processing instances that can be distributed across several servers. This coordination allows a potential elastic load balancing by launching multiple instances of the data processing software during peak sampling periods of the field season or where there may be many concurrent users.

### Trait extraction from LiDAR

The LiDAR data provides a 3D representation of the canopy which can be processed in multiple ways, enabling the extraction of multiple measurements from the same original raw data. In this manuscript, we focus on three key traits that are relevant in breeding, agronomy and crop physiology field experiments: canopy height, ground cover and above-ground biomass.

#### Canopy height

**Figure 4:**
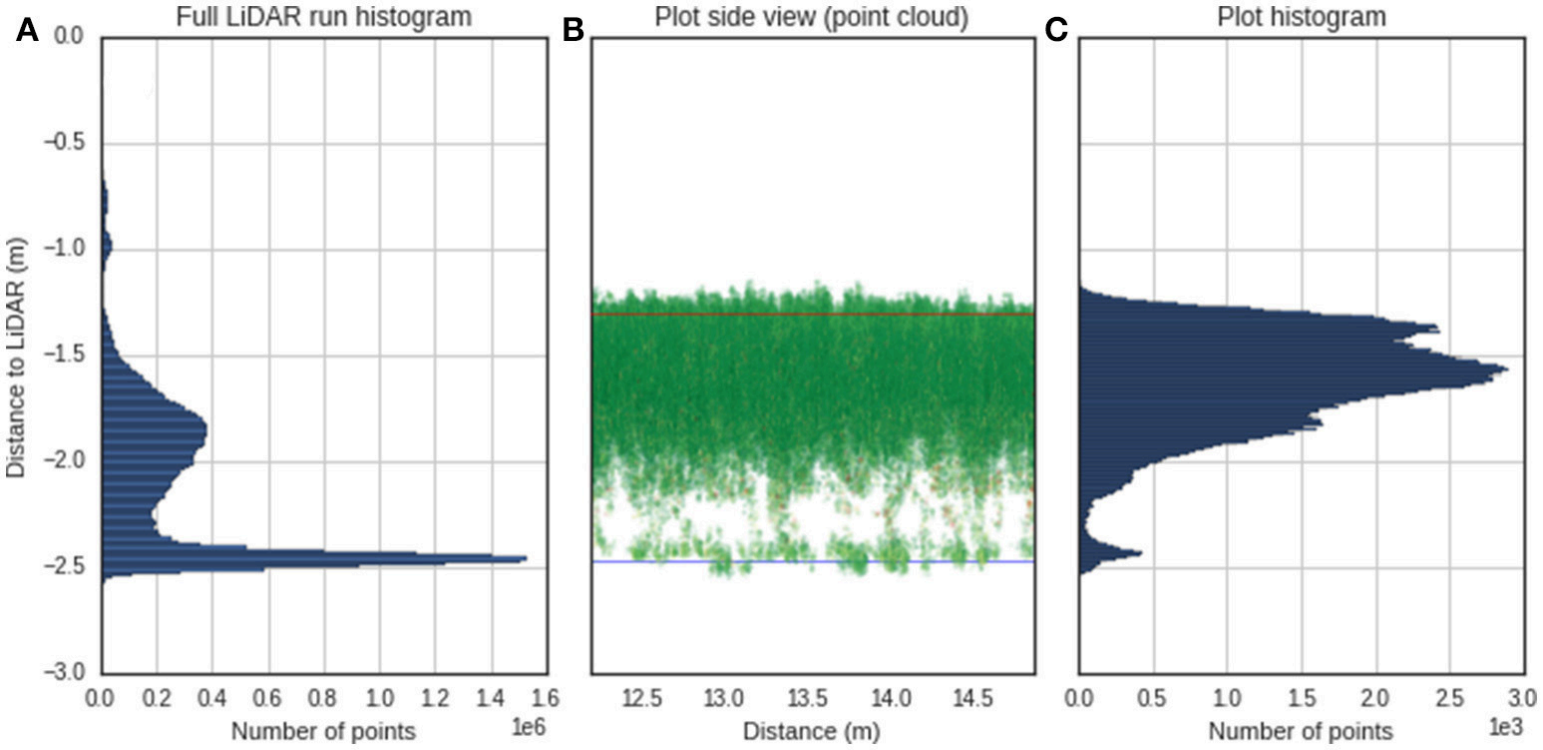
Determination of canopy height from LiDAR. **(A)** Histogram of distance relative to the LiDAR for a typical column of plots on the 23/10/2014. The peak of the histogram is selected as the height of the ground. **(B)** Side view of point cloud for a single plot from the same column as **(A)**, where the x-axis is the distance traveled by the Phenomobile Lite along the column. The blue line represents the ground obtained as the peak of the histogram, and the red line represents the height for the 0.955 quantile. **(C)** Histogram for the same single plot as **(B)** where the user has selected the region of interest.

Determining the canopy height with LiDAR requires estimation of the ground elevation and subtracting this from the absolute height of the points. Most of the published methodologies are based on the determination of a crop surface model and a digital terrain model, where the difference provides the crop height (Louise Loudermilk et al., 2009). The digital terrain model can be obtained from a scan with bare soil, while the crop surface model is calculated from the topmost points of the point cloud, using a selection based on a top percentile (Hämmerle and Höfle, 2014; Friedli et al., 2016). For the Phenomobile Lite, the nominal distance from the LiDAR sensor to the ground is fixed; therefore, it could be measured manually or automatically determined from the LiDAR. This provides a relative coordinate system where the ground elevation is always known. To avoid the manual measurement of the LiDAR position, the ground elevation for a given column of plots was assumed as the peak of the histogram (i.e., mode) of heights in the point cloud (Figure 4A). The prominence of this peak is evident in Figure 4A and arises because a column regularly includes sections of space between plots with bare soil. For a given experimental plot (Figures 4B,C), canopy height is estimated as the difference between the ground elevation and a quantile value in the *z* coordinate. To determine the optimum quantile defining the top of the canopy, quantile values ranging from 0.8 to 1.0 at increments of 0.005 were tested against manual height measures with a ruler. The root mean square error (RMSE) was calculated between LiDAR canopy height and canopy height measured manually.

#### Canopy ground cover

We evaluated two LiDAR algorithms for the classification of vegetation and soil to derive GC. In both cases, the LiDAR point cloud is transformed into a raster image that then is evaluated for the estimation of GC.

##### Images generated from the red reflectance of the LiDAR

**Figure 5:**
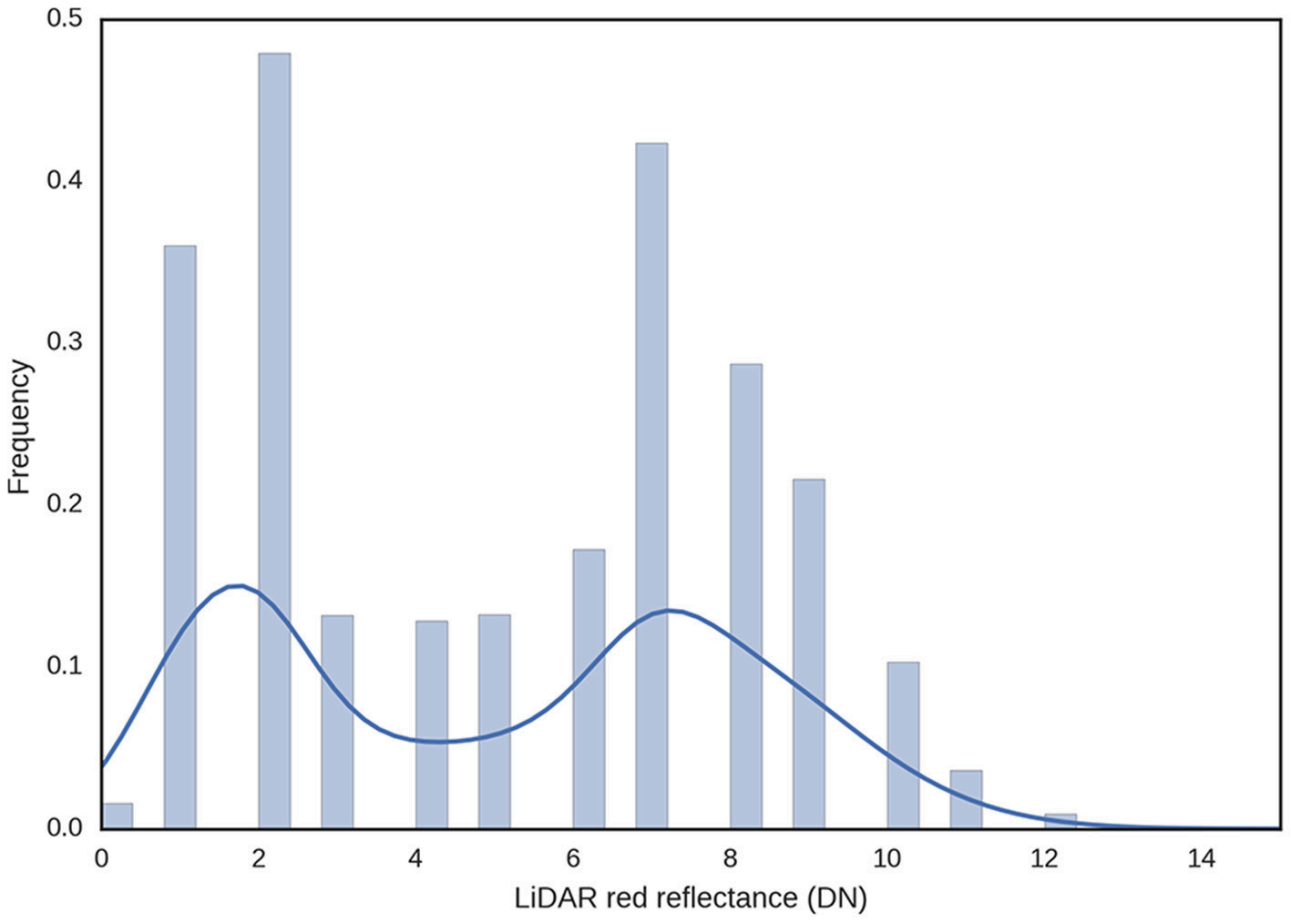
Frequency distribution of LiDAR red reflectance for a typical experimental plot. Green tissue from plants will tend to absorb the red light, while soil will tend to reflect the red light. GC was estimated using a binary rule, where LiDAR intensity less than five was classified vegetation and LiDAR intensity five and above was classified as soil.

**Figure 6:**
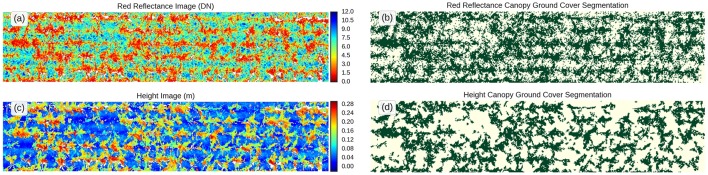
Canopy ground cover determination. Top views of the same experimental plot. False color scale for **(a)** red reflectance and **(c)** height. Binary images showing estimation of canopy ground cover after canopy segmentation using threshold values for **(b)** reflectance (<5) and **(d)** height (10 cm).

The LiDAR's red laser enables discrimination of plants from soil based on the assumption that green tissue from plants will absorb most of the red light, while the soil will have a higher reflectance in the red region of the spectrum. Therefore, the histogram analysis of the red reflectance from the LiDAR typically shows two distinct peaks for the vegetation and soil (Figure 5). This was used to estimate GC whereby LiDAR intensity less than five was classified as vegetation and LiDAR intensity of five and above was classified as soil (Figures 6a,b).

##### Height analysis

Since the LiDAR provides a 3D representation of the canopy, any organ or tissue above the ground can be considered vegetation and therefore, the calculation of GC can be derived from that classification (Figures 6c,d). This method may be suitable when the canopy is senesced, with little green tissue, or if the soil is dark. However, it may be unreliable during early crop stages where the height of the canopy is too close to the ground or when plants are grown in deep furrows.

For the validation of the LiDAR-derived GC, RGB digital color images were acquired with the Canon 6D DSLR camera mounted on the Phenomobile Lite. The camera was triggered automatically based on distance, and one image was taken every 1 m. The focus was fixed, and camera settings were set to manual with fast exposure and high ISO for minimizing any motion blurring. The camera integrates an internal GPS that is configured to set the internal clock in sync with the GPS time. Since the internal GPS update rate is 1 s and does not have DGPS capabilities, there was not enough accuracy for geo-locating the RGB images to the plots. Instead, we used the GPS timestamp for each image and the GPS-IMU track. Once the plots have been extracted in the data processing pipeline it is possible to attribute each RGB image to a different plot. The result is multiple RGB images per plot, depending on the length of the plots. The RGB images were analyzed using the methodology and software developed in Li et al. (2010). The software imports the RGB images and estimates the portion of green pixels based on the SAVI_green_ vegetation index (Huete, 1988) for each image. The mean GC of each plot was calculated averaging the images belonging to the plot. We also used NDVI measurements acquired simultaneously with a GreenSeeker sensor mounted on the Phenomobile Lite. The raw NDVI measurements from the GreenSeeker were registered using a serial port (RS232) and integrated with the GPS-IMU track. The NDVI measurements were averaged at the plot level using the position and plot location.

#### Above-ground biomass

Two LiDAR methods for the estimation of above-ground biomass were evaluated:

##### (a) Voxel-based method

The 3D box containing all the points for a given experimental plot were subdivided into voxels of regular dimensions (height = width = length), creating a 3D grid with the number of elements being a function of the total size of the 3D box for the experimental plot and the voxel size. Only the points above a 10 cm ground offset were included in the analysis. The ground elevation was calculated using the same methodology that was used for the canopy height. The coordinates of each point in the point-cloud were checked to allocate each point to its respective voxel. The resulting 3D grid, containing the number of points within each voxel, was filtered to eliminate voxels with less than 10 points as a way to remove spurious points and outliers that may not have been eliminated by the cleaning algorithm. The ratio of the number of voxels containing points to the number of subdivisions in the horizontal plane (width × length) was calculated and herein referred to as the 3D Voxel Index (3DVI). The 3DVI was calculated for voxel sizes ranging from 10 mm to 200 mm, at 10 mm increments. To determine the optimal voxel size, the RMSE and coefficient of determination (*r*^2^) were determined for the linear regression between 3DVI, at the given voxel size, and above-ground biomass measured manually.

##### (b) Profile-based method

The point-cloud for a given experimental plot was divided into vertical layers of 10 mm and, for each layer, the fraction of points divided by the total number of points for the point-cloud was calculated. Then, starting from the top, each layer was corrected by a factor, analogous to the extinction coefficient in Beer's law (Richardson et al., 2009), calculated as the exponential of the correction factor (*k*), multiplied by the total fraction of points intercepted above that layer. The sum of the corrected fraction of points for each layer was calculated and herein referred to as the 3D Profile Index:

(1) $$\begin{array}{l} 3DPI=\sum_{i=0}^{i=n}\left(\frac{p_{i}}{p_{t}}e^{k\frac{p_{cs}}{p_{t}}}\right) \end{array}$$

**Figure 7:**
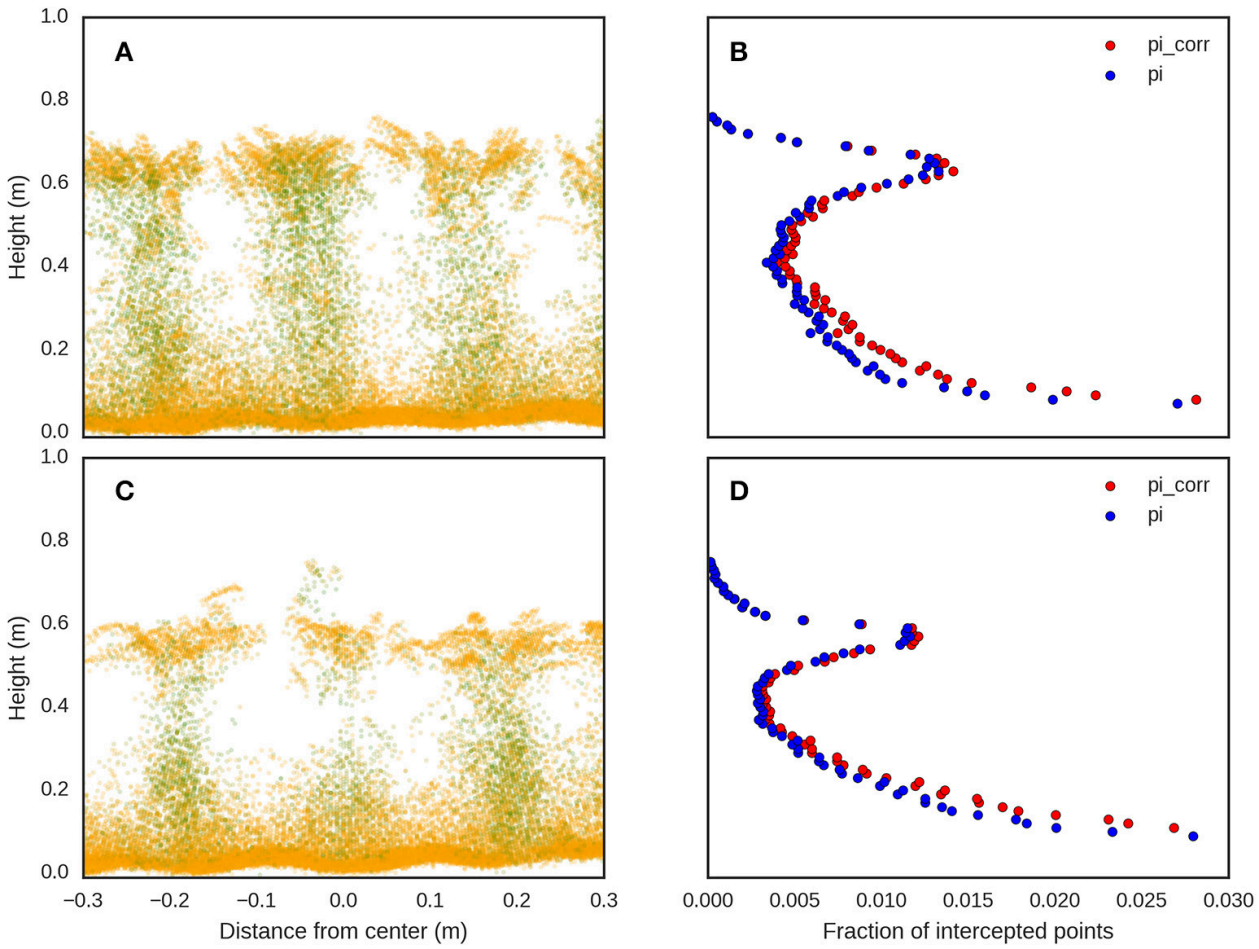
LiDAR point clouds, representing the cross sections for two different experimental plots, **(A,C)**, from the same experiment with contrasting genotypes and different above-ground biomass at maturity (8th December 2015) of 12.0 and 7.2 t/ha for **(A,C)** respectively. The corresponding fraction of intercepted points (*p*_*i*_) and corrected intercepted points (*p*_*i*_*corr*_) with *k* = −1, for **(A,C)**, are shown in **(B,D)** respectively.

Where: *i* is a given 10 mm vertical layer with 0 and n the lower and uppermost layers respectively; *p*_*i*_ is the LiDAR points for a given layer; *p*_*t*_ is the total LiDAR points for all layers; *p*_*cs*_ is the cumulative sum of LiDAR points intercepted above a given layer. Figure 7 shows an illustrative example of the profile-based method. The optimal value of *k* was determined by calculating 3DPI, for *k* values ranging from −3.5 to 2.25 at increments of 0.05, and determining the RMSE and coefficient of determination between 3DPI, at the given *k* value, and above-ground biomass measured manually. A 10 cm ground offset was used to determine the lowest layer and the ground elevation was calculated using the same methodology that was used for the canopy height.

### Field experiments

We conducted three separate experiments for the validation of canopy height, canopy ground cover, and above-ground biomass using the Phenomobile Lite. Canopy height was validated in Experiment 1 (EXP1), canopy ground cover was validated in Experiment 2 (EXP2) and above-ground biomass was validated in Experiment 3 (EXP3).

#### (a) Experiment 1: validation of canopy height

EXP1 comprised a selection of 18 near-isogenic wheat lines, arising from mutagenesis of the Brazilian bread wheat cultivar Maringá, with allelic variants of the *Rht-B1* allele and therefore known phenotypic variation for height (Chandler and Harding, 2013). The experiment was sown on 12th June 2014, at Yanco NSW (34.62S, 146.43E, elevation 164 m) in SE Australia, and comprised three replicate experimental plots per genotype (54 experimental plots in total) sown in a randomized complete block design (orientated North-South). Experimental plots were 12 m long and 10 rows across with a row spacing of 0.18 m and sowing density of 250 seeds/m^2^.

Phenomobile Lite measurements with LiDAR were made on the 23rd Oct. 2014. On the same day, canopy height was measured manually following Rebetzke et al. (2013b), with three replicate measures of canopy height per experimental plot.

#### (b) Experiment 2: validation of canopy ground cover

EXP2 comprised 90 entries of commercial and advanced breeder lines and was sown on the same date and location as EXP1 into a randomized complete block design with three replicate experimental plots per genotype (270 experimental plots in total and orientated North-South). Experimental plots were 6 m long and 10 rows across with a row spacing of 0.18 m and sowing density of 250 seeds/m^2^.

Phenomobile Lite measurements, including LiDAR, GreenSeeker and Canon 6D DSLR camera for validation of GC, were made across a range of phenological growth stages, ranging from early tillering to grain-filling, on the following five dates: 13 Aug. 2014; 21 Aug. 2014; 8 Sept. 2014; 16 Sept. 2014; 23 Sept. 2014.

#### (c) Experiment 3: validation of above-ground biomass

Thirteen contemporary bread wheat (*Triticum aestivium* L.) and two triticale (*x Tricosecale*) genotypes were sown on the 12th June 2015 at the CSIRO Agriculture and Food Ginninderra Experiment Station (GES), Canberra, ACT, Australia (35.20S, 149.09E, elevation 577 m). The genotypes were selected to represent a broad range of canopy architecture, including very erect to prostrate, and thereby enable robust evaluation of the LiDAR for phenotyping. The experimental plots were 15 m long and 10 rows across with a row spacing of 0.18 m, allowing for multiple destructive assessments of above-ground biomass. Sowing density was 250 seeds/m^2^ and in five genotypes, an additional low-density treatment (125 seeds/m^2^) was added to increase the range of above-ground biomass. The experiment comprised 60 experimental plots in total (three replicates of 15 genotypes sown at 250 seeds/m^2^ and five genotypes sown at 125 seeds/m^2^), orientated North-South and sown in a randomized complete block design.

Eight destructive above-ground biomass sampling events were undertaken at different phenological stages from tillering to maturity. Plant development stage was recorded at each above-ground sampling event, using the Zadoks development scale (Zadoks et al., 1974; Table 1). Above-ground biomass was determined from shoots cut at ground level from the central six rows of each plot along 1.0 m in length (sample area of 1.08 × 1.0 m = 1.08 m^2^). The dry weight was determined from the samples after drying at 65°C until reaching a constant dry weight. LiDAR measurements with the Phenomobile Lite were obtained on the same day or immediately prior to an above-ground biomass sampling event. For the LiDAR analysis, we selected the section of the plot where the above-ground biomass sampling was going to be performed (ca. 1.0 m^2^).

**Table 1:** Date and Zadoks growth stage (average for all the plots) for the eight above-ground biomass sampling events for EXP3 at Ginninderra Experiment Station, Canberra ACT in 2015.

**Date**	**Zadoks**	**Stage**
2015/09/11	Z21	Tillering
2015/09/24	Z31	Stem elongation
2015/10/07	Z32	Stem elongation
2015/10/14	Z42	Flag leaf visible
2015/10/23	Z55	Head emergence
2015/10/30	Z65	Anthesis
2015/11/23	Z85	Grain-filling
2015/12/08	Z95	Maturity

*Above-ground biomass samples were of 1.08 × 1.0 m dimension and Phenomobile Lite measurements were made on the same day and immediately prior to an above-ground biomass sampling event*.

## Results

### Canopy height

**Figure 8:**
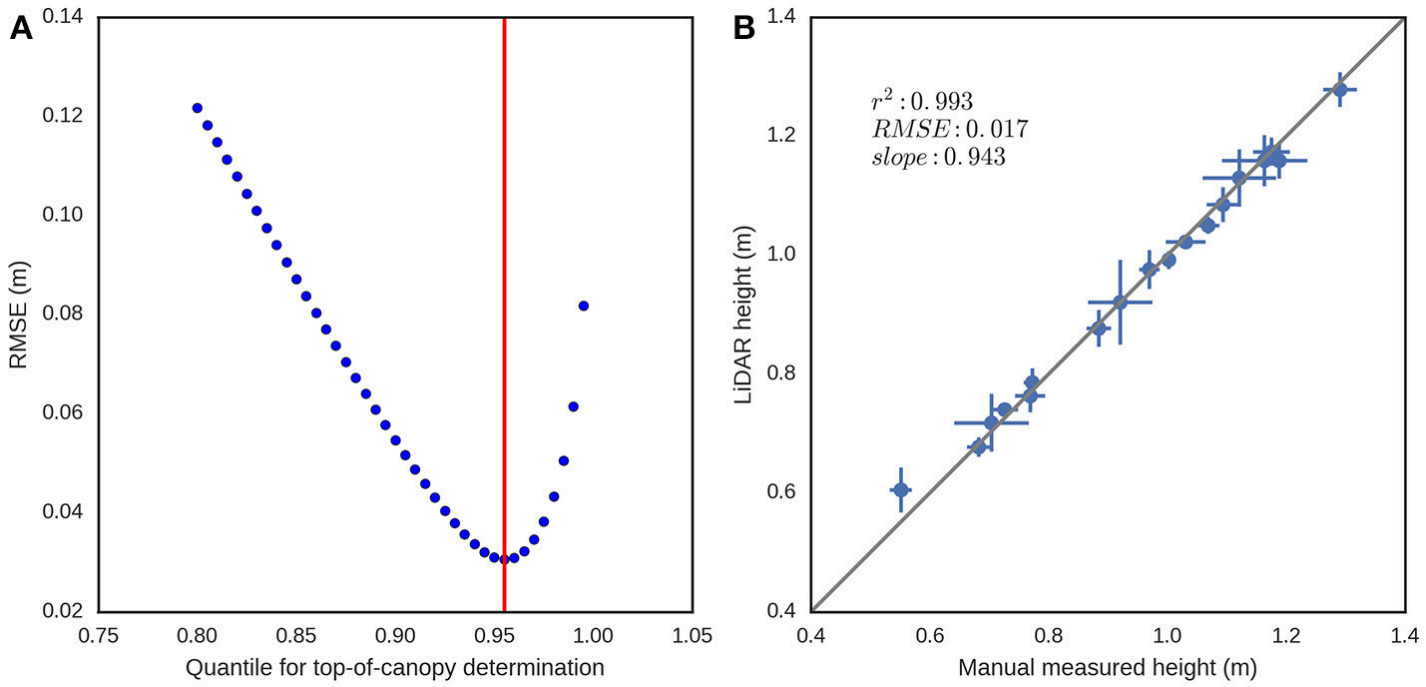
Canopy height validation from EXP1 on the 23rd Oct. 2014. **(A)** RMSE between canopy height measured manually with three replicate measures of canopy height per experimental plot and LiDAR canopy height derived from different quantile values, ranging from 0.8 to 1.0 at increments of 0.005, in the *z* coordinate. **(B)** Scatter plot of canopy height measured manually and LiDAR canopy height for the optimum quantile of 0.955. Error bars are plus-minus the standard deviation of the measurements for each genotype (three replicate experimental plots per genotype and 18 genotypes).

The results from the canopy height validation (EXP1) are shown in Figure 8. The optimum quantile was 0.955, as determined by the smallest RMSE between measurements made on 23rd Oct. 2014 of canopy height measured manually and LiDAR canopy height, derived from quantile values ranging from 0.8 to 1.0 at increments of 0.005 in the *z* coordinate (Figure 8A). The coefficient of determination (*r*^2^) and RMSE between canopy height measured manually and LiDAR canopy height, with data aggregated by genotype (Figure 8B) was 0.993 and 0.017 m respectively (data from 23rd Oct. 2014), with a slope of 0.943.

### Canopy ground cover

**Figure 9:**
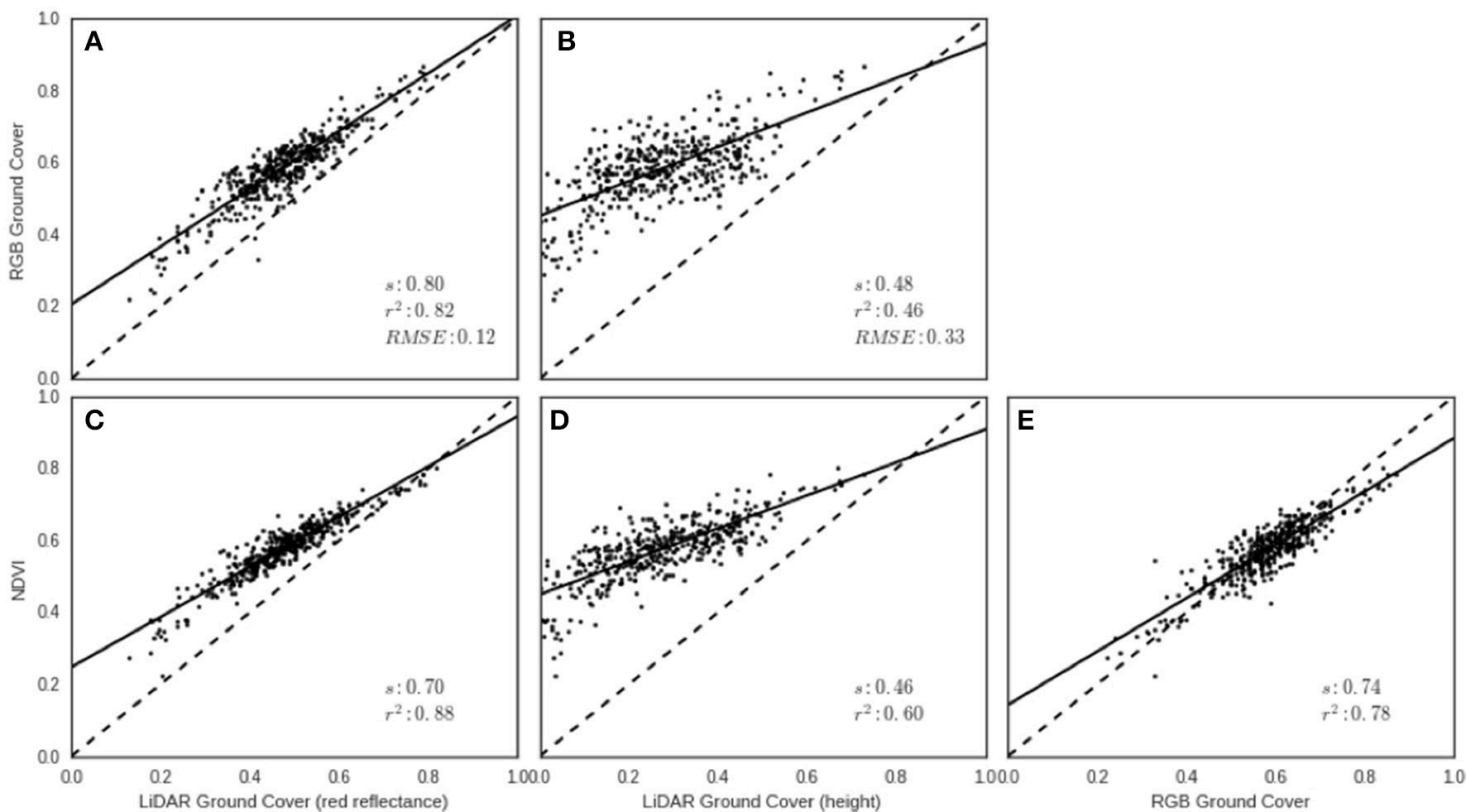
Ground cover (GC) validation from EXP2 on 13th August 2014. Relationships between LiDAR-derived GC using **(A)** red reflectance and **(B)** height with RGB-derived GC. Relationships between LiDAR-derived GC using **(C)** red reflectance and **(D)** height, with NDVI from the GreenSeeker. Relationship between **(E)** RGB-derived GC and NDVI from the GreenSeeker. Solid lines represent the linear regression and broken line represents 1:1.

Canopy ground cover (GC) estimates derived from the LiDAR using red reflectance and height were compared with GC derived from the RGB images using the protocol described in Li et al. (2010), for each experimental plot in EXP2 on 13th August 2014. LiDAR red reflectance GC was strongly associated with RGB GC (Figure 9A, *r*^2^ = 0.82), and NDVI (Figure 9C, *r*^2^ = 0.88), but the LiDAR red reflectance tended to underestimate GC compared with the RGB camera (slope = 0.80) and NDVI (slope = 0.70). The association between NDVI and RGB GC was also strong (Figure 9E, *r*^2^ = 0.78). The LiDAR height GC resulted in the smallest coefficient of determination values between RGB GC (Figure 9B, *r*^2^ = 0.46) and NDVI (Figure 9D, *r*^2^ = 0.60). The LiDAR height method clearly underestimated GC when compared to the values obtained from the RGB images and NDVI.

**Figure 10:**
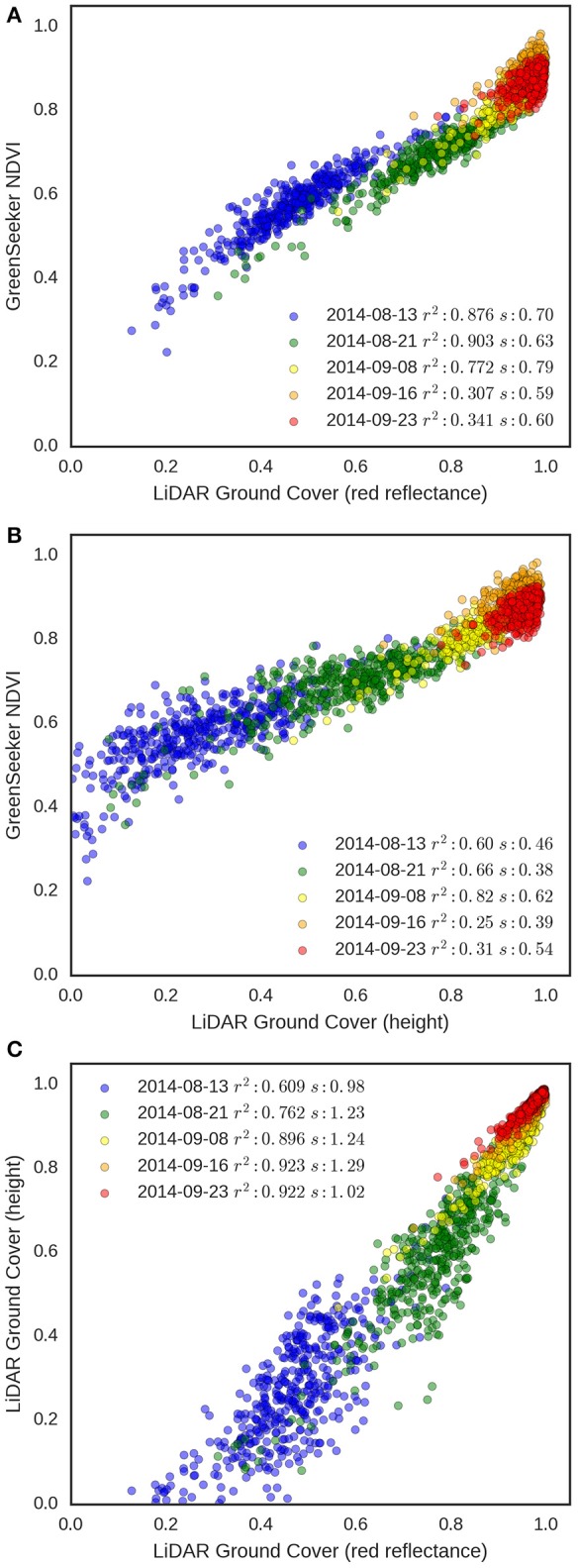
GC validation from EXP2. Relationships between NDVI from GreenSeeker and **(A)** LiDAR red reflectance GC and **(B)** LiDAR height GC. **(C)** Relationship between the two LiDAR GC methodologies. Data points are for each experimental plot (*n* = 270 in EXP2) on five different dates (13 Aug. 2014, 21 Aug. 2014, 8 Sept. 2014, 16 Sept. 2014, 23 Sept. 2014). For each date, *r*^2^ and slope (s) of the linear regression are shown.

To evaluate the robustness of the GC methodologies as the crop develops, GC derived from the LiDAR using both red reflectance and height-based methods were compared to GreenSeeker NDVI measurements for each experimental plot in EXP2 on five different occasions from tillering to head emergence (Figure 10). The results show a strong association between LiDAR GC and NDVI in the early developmental stages with coefficients of determination ranging from 0.77 to 0.90 for the reflectance method and 0.60 to 0.82 for height, for the first three dates. However, for the final two dates, the range of NDVI and GC decrease and the association with NDVI weakens for both methodologies, with *r*^2^ ranging from 0.25 to 0.34. This suggests that NDVI saturates at GC values above 0.8 as previously reported (Prabhakara et al., 2015). The association between the two LiDAR GC methodologies was highly linear (Figure 10C) and the *r*^2^ progressively increased from 0.61, for the first run on the 13th August 2014, to 0.92 by canopy closure. However, the height method tended to underestimate GC when compared with the reflectance method, especially at the earlier dates.

### Above-ground biomass

**Figure 11:**
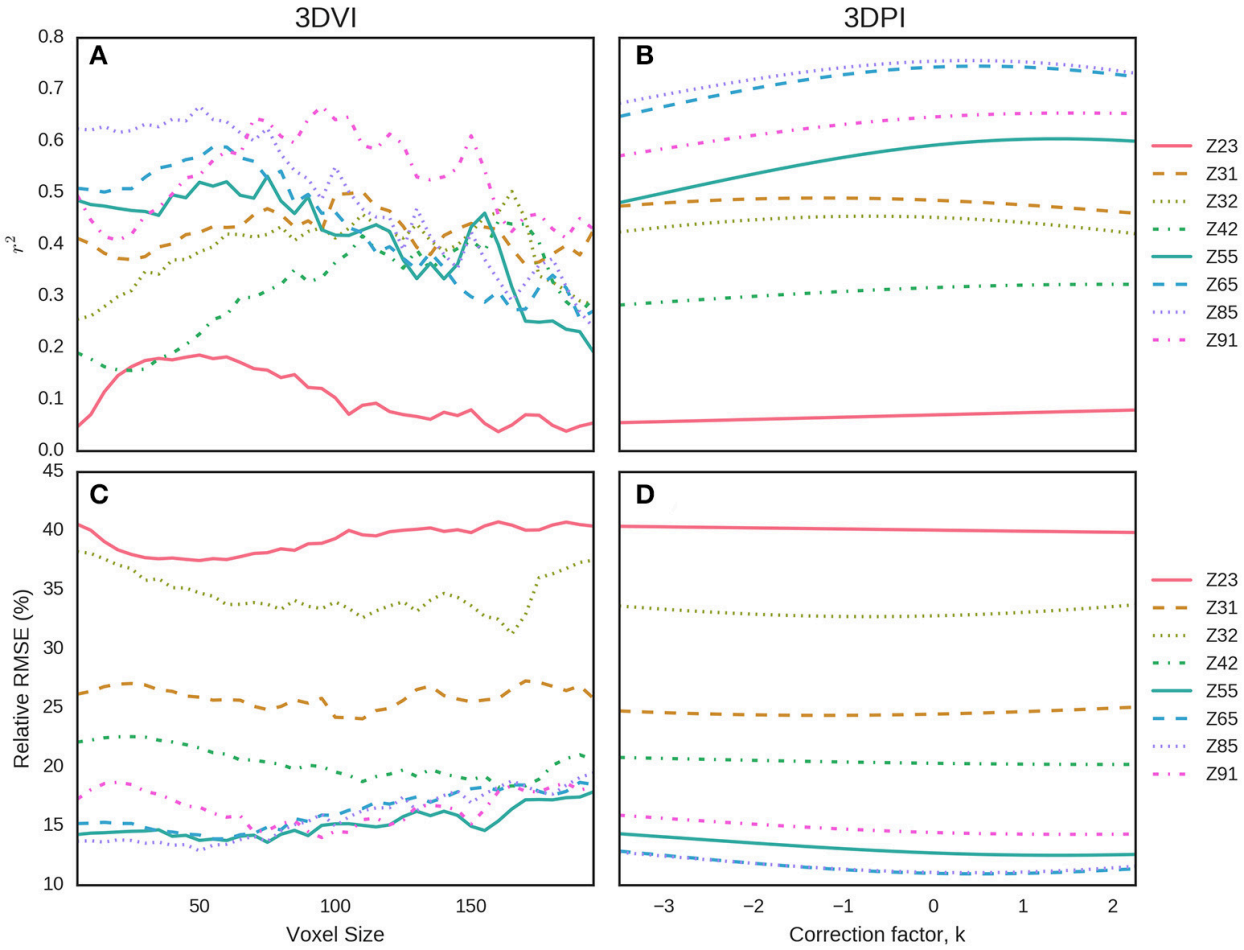
Determination of the optimum parameters for 3DVI (voxel size) and 3DPI (k) for the estimation of above-ground biomass from EXP3 at eight sample events. For each parameter value, the *r*^2^ **(A,B)** and relative RMSE (%) **(C,D)** of the linear regression with above-ground biomass were calculated. For 3DVI, voxel sizes ranged from 10 to 200 mm, at 10 mm increments **(A,C)**. For 3DPI, *k* ranged from −3.5 to 2.25 at increments of 0.05 **(B,D)**.

Data from EXP3 was used to evaluate and compare the two LiDAR methodologies presented here for estimating above-ground biomass. We evaluated the effects of the key parameters, namely the voxel size for the 3DVI and the correction factor, *k*, for the 3DPI, to determine the optimum values across the different sample events (Figure 11). In both cases, the indices were calculated for a range of parameter values (voxel sizes from 10 to 200 mm, at 10 mm increments, and *k* from −3.5 to 2.25 at increments of 0.05). For each parameter value, at each sampling event, the RMSE and *r*^2^ were calculated for the linear regression between 3DVI, 3DPI, and the field measurements of above-ground biomass. For the 3DVI, the voxel size had a strong effect on the *r*^2^ and RMSE for all sample dates, with *r*^2^ values ranging from 0.0 to 0.64 depending on the sample date and voxel size. 3DPI was less sensitive to changes in the k parameter for each date and changes only marginally affected the coefficient of determination, thereby providing more stable above-ground biomass estimates across the evaluated range of k.

A summary of the optimal parameters that maximized *r*^2^ and minimized RMSE for each date, overall, pre-anthesis and post-anthesis are presented in Table 2. Both 3DVI and 3DPI performed poorly at earlier sample dates but improved as the crop evolved. From 23 Oct 2015 (~Z55), *r*^2^ was greater and RMSE lower for 3DPI, compared with 3DVI on all the single dates, except at Z91 when 3DVI was slightly superior to 3DPI (*r*^2^ = 0.67 vs. *r*^2^^.^ =^.^0.65). For both methods, the highest *r*^2^ and lowest RMSE were obtained on 23 Nov. (~Z85), with *r*^2^ = 0.67, RMSE = 12.89% and *r*^2^ = 0.76, RMSE = 11.04% for 3DVI (voxel size = 50 mm) and 3DPI (*k* = 0.50) respectively. When combining all the sample events [Equations (2, 3)], *r*^2^ increased to 0.81 for 3DVI and 0.73 for 3DPI, but the RMSE also increased to 31.65 and 38.25% respectively. Two relationships were calculated for before and including anthesis [Z ≤ 65, Equations (4, 5)] and post-anthesis [Z > 65, Equations (6, 7)]. For Z ≤ 65 both indices performed similarly (*r*^2^ = 0.86 and *r*^2^ = 0.85, RMSE = 24.10% and RMSE = 25.43% for 3DVI and 3DPI respectively), whereas for Z > 65 3DPI outperformed 3DVI (*r*^2^ = 0.68, RMSE = 13.33% vs. *r*^2^ = 0.54, RMSE = 16.06%).

**Table 2:** Summary of maximum r^2^ and minimum RMSE (%) and corresponding optimum voxel size, for 3DVI, and *k*, for 3DPI, for the linear regression between the corresponding index and above-ground biomass measured in EXP3 for each sample event shown in Figure 11.

		**3DVI**			**3DPI**		
**Sample date**	**Zadoks development stage**	**Optimum voxel size (mm)**	**RMSE (%)**	**r^2^**	**Optimum *k***	**RMSE (%)**	**r^2^**
2015-09-11	Z23	50	37.48	0.19	2.25	34.99	0.02
2015-09-24	Z31	110	24.07	0.50	−1.50	23.46	0.48
2015-10-07	Z32	165	31.26	0.50	−0.75	32.73	0.45
2015-10-14	Z42	160	18.30	0.44	2.0	20.21	0.32
2015-10-23	Z55	75	13.61	0.53	1.50	12.52	0.60
2015-10-30	Z65	55	13.90	0.59	0.50	10.94	0.74
2015-11-23	Z85	50	12.89	0.67	0.50	11.04	0.76
2015-12-08	Z91	95	14.04	0.67	1.50	14.29	0.65
Overall	All	130	31.65	0.81	−1.50	38.25	0.73
Overall pre-anthesis	≤Z65	80	24.10	0.86	−0.50	25.43	0.85
Overall post-anthesis	>Z65	75	16.06	0.54	0.75	13.33	0.68

*Optimum parameters are also calculated for all the sampling dates (overall), pre-and including anthesis and post-anthesis*.

**Figure 12:**
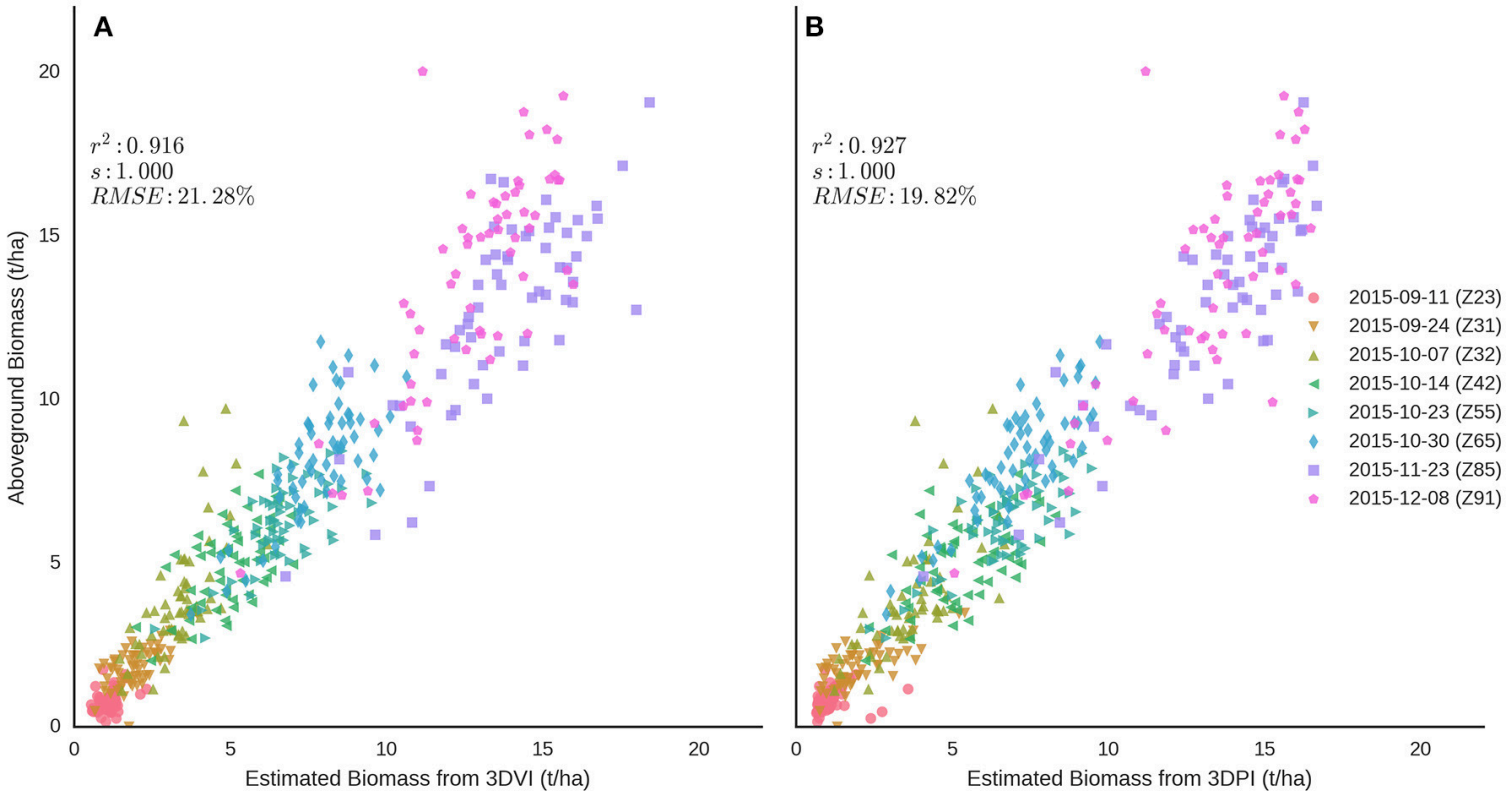
Relationships between above-ground biomass and LiDAR estimations of biomass from **(A)** 3DVI and **(B)** 3DPI, using Equations (4, 6) for 3DVI and [5] and [7] for 3DPI. Each equation was applied across multiple sample dates according to the nominal phenological stage of each sampling date (i.e., Z ≤ 65 or Z > 65). Refer to Table 2 for the optimal voxel size or *k*, for 3DVI and 3DPI respectively (i.e., Z ≤ 65 or Z > 65). Sampling dates are represented with different colored symbols.

Above-ground biomass was estimated using a combination of Equations (4, 6) (Figure 12A) for 3DVI and Equations (5, 7) for 3DPI (Figure 12B). In each case, the equations and the optimal voxel size or *k*, for 3DVI and 3DPI respectively, shown in Table 2, were used according to the approximate phenological stage (i.e., Z ≤ 65 or Z > 65). The results suggest a strong relationship across the sample dates between estimated biomass and manual measurements, with better results for 3DPI [*r*^2^ = 0.927, RMSE = 19.82% (1.30 t/ha)] compared with 3DVI [*r*^2^ = 0.916, RMSE = 21.28% (1.39 t/ha)].

(2) $$\begin{array}{lll} Biomass_{all} & = & 2.18949\cdot 3DVI-1.26579 \end{array}$$

(3) $$\begin{array}{lll} Biomass_{all} & = & 6.97721\cdot 3DPI+0.69980 \end{array}$$

(4) $$\begin{array}{lll} Biomass_{pre} & = & 1.05604\cdot 3DVI+0.34608 \end{array}$$

(5) $$\begin{array}{lll} Biomass_{pre} & = & 7.66505\cdot 3DPI+0.65564 \end{array}$$

(6) $$\begin{array}{lll} Biomass_{post} & = & 1.33361\cdot 3DVI+2.96524 \end{array}$$

(7) $$\begin{array}{lll} Biomass_{post} & = & 26.4468\cdot 3DPI-1.42782 \end{array}$$

## Discussion

Field phenotyping still remains a bottleneck in the pipeline of high throughput phenotyping (Araus and Cairns, 2014), where limited options are readily available for performing measurements of physiological traits at a large scale (Furbank and Tester, 2011). The Phenomobile Lite was designed for routine operation in large field experiments and breeding trials and deployment in such applications has clear advantages over current practice. For example, the Phenomobile Lite is easily transported to the field, thereby overcoming a major limitation of fixed phenotyping platforms where experiments are constrained to their occupied space (Kirchgessner et al., 2017; Virlet et al., 2016). When operated at walking speed, the Phenomobile Lite can measure multiple traits simultaneously on ~800 10 m^2^ plots/h and measurements can be repeated at different developmental stages. The simple operation of the platform ensures that non-technical users can operate the instrument, and its design is amenable to automation and autonomous navigation in future versions. Further, the Phenomobile Lite is modular, enabling the integration of multiple sensors. Herein we demonstrate the integration of a digital RGB camera and an active NDVI sensor that operate in coordination with the LiDAR using a standard spatial reference provided by a GPS/IMU and present and validate LiDAR-based algorithms for providing high-throughput non-destructive estimates of canopy height, ground cover and aboveground biomass.

### High accuracy of LiDAR canopy height

The low RMSE of 0.017 m (*r*^2^: 0.993, slope: 0.943), between LiDAR canopy height and canopy height measured manually was consistent with other reported accuracies such as 0.018 m in wheat (Virlet et al., 2016), 0.024 m in triticale (Busemeyer et al., 2013), ~0.03–0.06 in barley (Tilly et al., 2015), ~0.05 m in rice (Tilly et al., 2014). Although estimating height from the LiDAR is an obvious use of this instrument, this still required determination of the top of the canopy and the ground elevation. The top of the canopy was determined by analyzing the frequency distribution of height from the LiDAR and using the optimum quantile of 0.955, as defined in the EXP1 validation. This optimum value is smaller than the value (0.99) obtained in Friedli et al. (2016), using a terrestrial LiDAR scanner (TLS), but it is close to the 0.95 quantile used originally in Deery et al. (2014) and greater than the 0.9 used in (Hämmerle and Höfle, 2014). The TLS used in Friedli et al. (2016) and (Hämmerle and Höfle, 2014) perform the scans from a single point, creating spheres of point clouds where the laser beam penetrates into the canopy from a tilt angle that is steeper as one gets away from the scanning point. As the laser beam will not penetrate much into the canopy after closure, most of the points will come from the top of the canopy, which could explain the higher quantile when compared with a line scanner system that scans from a nadir perspective. In this study, the ground elevation was determined for a given experimental column from the bare soil between experimental plots. For closely-spaced experimental plots with little to no bare soil in-between, the estimation of ground elevation may fail and would require manual specification of the distance from the LiDAR to the ground. Most published methods for the estimation of canopy height from LiDAR or aerial photography, are based on the determination of crop surface models (Hoffmeister et al., 2013), which require determining the ground elevation from a scan with bare soil. In this case, the fixed geometry of the LiDAR with respects to the ground makes this step unnecessary. Determination of the top of the canopy assumes a uniform canopy, which may not be the case for plots with poor establishment or lodging. In these cases, given the large number of sampling points for canopy height, the plot could be subdivided into smaller areas where the presented algorithm is applied. This could provide a measurement of the plot uniformity with the statistical distribution of the plot height, which could lead to an indicator of the plot health or lodging score.

### Relationships between LiDAR-based ground cover, NDVI and RGB-based ground cover

Canopy GC is an important trait in wheat, relevant in early developmental stages for both enhancing water-use efficiency through minimizing water loss through soil evaporation and for maximizing canopy light interception (Fischer, 1981; Richards and Rebetzke, 2002; Rebetzke et al., 2004; Mullan and Reynolds, 2010). Digital RGB images and NDVI are commonly used for quantifying GC and can provide relatively high-throughput (Rebetzke et al., 2013a). However, there are potential issues with both approaches. As a passive sensor, RGB imaging could be adversely impacted by the light conditions (over and underexposure) and, as the crop develops, classifying the green vs. non-green pixels could also be problematic because of shadowing from the canopy or senescence (Yu et al., 2017). The GC measurements made using active NDVI sensors, such as the GreenSeeker® (Trimble, USA), are not impacted by light conditions but they can be negatively impacted by the soil reflectance (Huete, 1988). Further, when used for the determination of GC, variation in canopy greenness can influence the measurement of NDVI, resulting in potentially reduced accuracy in the presence of different nitrogen status or during the onset of senescence (Hansen and Schjoerring, 2003).

In this paper, we have shown that LiDAR can be used in two different ways to determine GC: (1) using red reflectance from the LiDAR's red laser to separate vegetation from soil; and (2) using height as a threshold to determine the vegetation above that height. These two approaches were tested and compared with GC estimated from RGB images and NDVI. The limitations for NDVI as mentioned earlier apply to the LiDAR red reflectance, namely varying soil reflectance and varying canopy greenness. For instance, a wet, dark soil could lead to low reflectance values that could be close to the threshold used for vegetation. In the case of vegetation, the onset of senescence or presence of chlorosis could lead to elevated red reflectance with values similar to the soil. The use of LiDAR height for GC avoids these two issues, but the approach is problematic when plants are small and their height is proximal to soil undulations such as furrows. A combination of both approaches depending on crop height could be employed to accurately measure ground cover from emergence through to maturity.

At the initial crop stages, GC estimated from LiDAR red reflectance presents a comparable alternative to RGB images or NDVI (Figures 9A,C). The use of RGB images for GC, whilst potentially simple and cost effective to acquire, still require image capture for each plot and data processing using existing image processing algorithms (e.g., Casadesús et al., 2007; Li et al., 2010). Both tasks require human intervention and are potentially prone to errors and subjectivity, which makes necessary the development of robust automatic segmentation algorithms and processing pipelines (Yu et al., 2017). The use of active sensors, such as LiDAR or GreenSeeker, have the potential advantage of reliability under a range of light conditions. For measurements collected from stem elongation to post-canopy closure, LiDAR height GC offers advantages over NDVI due to the absence of signal saturation. This saturation was evident in the plateau in NDVI that occurred around anthesis in EXP2 (23 Sept. 2014; see Figure 10), similar to the evolution of NDVI reported elsewhere. For example, Rebetzke et al. (2016) reported the evolution of NDVI and LiDAR, in relation to canopy stay green, from pre-anthesis to maturity, where NDVI reached a plateau pre-anthesis and decreased during grain-filling. In this case, the alternative of using LiDAR height GC avoids this issue and can still provide an estimate of ground cover with non-green vegetation.

### LiDAR predictions of biomass were strongly associated with above-ground biomass

The capacity to non-destructively estimate above-ground biomass using LiDAR is a critical outcome of this work, given the lack of rapid and non-destructive alternatives, especially after canopy closure in wheat. An additional motivating factor for this work was to overcome the limitations of NDVI for estimating aboveground biomass, namely, its saturation after canopy closure and the confounding influences of canopy greenness and soil reflectance. We tested two different algorithms for the determination of aboveground biomass from LiDAR. The first approach tested (3DVI), was based on the estimation of volumetric quantification of the above-ground biomass. This was performed through voxelization of the LiDAR point cloud and counting the number of voxels occupied by the canopy. For the second approach (3DPI), the vertical distribution of the LiDAR returns through the canopy were analyzed to develop a canopy density profile. That profile was integrated, resulting in the fraction of LiDAR points intercepted by the canopy.

Both 3DVI and 3DPI were strongly correlated with above-ground biomass after and including spike emergence (Figure 12), thereby overcoming limitations of using NDVI post canopy closure (Huete, 1988; Hansen and Schjoerring, 2003). The 3DPI outperformed the 3DVI after spike emergence (Z55, 23 Oct 2014), reaching a maximum coefficient of determination of 0.76 and minimum RMSE of 11.04% during grain-filling. Optimum parameters were also determined for all the data points across all the sample events resulting in a strong association (*r*^2^ = 0.81 for 3DVI and *r*^2^ = 0.73 for 3DPI) but also increased RMSE (>30%). The determination of combined optimum parameters for the samplings before and after anthesis revealed that prior to anthesis both indices performed similarly (*r*^2^ > 0.85 and RMSE <26%), but after anthesis 3DPI provided superior results (*r*^2^ = 0.68 vs. *r*^2^ = 0.54). Aggregating the data from all sample events pre and including anthesis and post-anthesis and applying the corresponding relationships [Equations (4–7)] to each of the sampling dates provided improved results, with slightly better results for 3DPI (*r*^2^ = 0.93, RMSE = 19.82%) when compared with 3DVI (*r*^2^ = 0.92, RMSE = 21.28%; Figure 12). These results are in line with previous studies which use relationships between height and biomass on wheat with *r*^2^ = 0.88 (Eitel et al., 2014) and rice with *r*^2^ = 0.9 (Tilly et al., 2014) or that combine height and vegetation indices with *r*^2^ = 0.84 (Bendig et al., 2015) and *r*^2^ = 0.85 (Tilly et al., 2015). The results provide evidence that the proposed method can operate over a broader range of dry biomass (up to 20 t/ha in this study vs. ~5t/ha Eitel et al., 2014; Tilly et al., 2015). Despite the considerable capacity of both 3DVI and, in particular, 3DPI, to quantify relative differences in above-ground biomass at full canopy closure, information about the phenological stage (pre and including anthesis or post anthesis) was still required to select the equations with the greatest prediction power.

The main limitation of both indices was their weak correlation with biomass at early growth stages. This problem is similar to the determination of GC using height. In both cases, a fraction of the canopy closest to the ground is ignored to avoid including rough terrain or furrows, created at sowing, thus contributing to an underestimation of above-ground biomass and GC at early growth stages. We feel that this limitation is minor, particularly as more meaningful physiological information from biomass comes from measurements taken from stem elongation to anthesis (e.g., Shearman et al., 2005). Destructive measurements taken at these stages are subject to many sources of error (e.g., the small subsection of the plot and the repeated handling of the samples during drying and weighing contribute to error) and cannot be taken at regular intervals; the LIDAR approaches overcome these issues, thereby providing greater confidence in the estimates obtained. It is possible however that an algorithm that separated plants from soil, by 3D reconstruction of the terrain for example, would potentially provide better estimations of above-ground biomass and GC at early growth stages to complement the promising results obtained here.

It is important to highlight that the LiDAR estimates of biomass are mainly driven by changes in the bio-volume of the canopy, as measured by the LiDAR sensor and then represented in the 3D point cloud. Despite the strong association of the proposed LiDAR indices with above-ground biomass, this method cannot explicitly account for changes in biomass resulting from remobilisation from the vegetative to the reproductive organs during grain-filling or even changes in tissue density. For example, a low final biomass resulting from biotic or abiotic stress during grain-filling may not be picked up by these indices, unless changes in the volume of the heads (i.e., an indirect measurement of grain size and grain number) become evident and detectable in the biovolume estimates or the 3D profile. Besides, the application of these methodologies in very dense canopies, where the upper layers intercept most of the points, preventing the laser beam to penetrate into the lower parts of the canopy, will present an underestimation of biomass and a poorer description of the canopy architecture from the LiDAR point cloud. These limitations may be overcome by using alternative sensor technologies that measure the water content or the canopy bulk density, which would help monitor the status of grain-filling or provide estimates of the density or plant organs. The potential synergies between LiDAR and hyperspectral imaging (Geipel et al., 2014; Bendig et al., 2015; Tilly et al., 2015) or microwave sensing could enable the development of new multi-sensor indices which would measure changes in the canopy density as it develops or provide an insight of the occluded layers in the canopy, providing more robust estimates of above-ground biomass.

## Conclusions

We have demonstrated the capacity for non-destructive and accurate high-throughput measurement of canopy height, ground cover and above-ground biomass in the field using LiDAR. The Phenomobile Lite, presented herein, was designed for simple operation and cost-effective use on large field experiments. The main sensor is the LiDAR, but the Phenomobile Lite can accommodate additional instruments including a GreenSeeker, for NDVI, and a RGB digital camera; other sensing platforms can be added in future. A custom-developed web interface was developed for data processing by the non-technical user, enabling rapid and simple data extraction.

The deployment of the Phenomobile Lite within genetics, physiology, and agronomy studies, or plant breeding programs will enable the non-destructive measurement of canopy height, GC, and above-ground biomass on a larger scale than typically undertaken, by overcoming the resource-intensive nature of manually measuring these traits. Further, sampling will encompass the entire area of the plot to reduce sampling and increase precision than previously with portions of the canopy. The non-destructive nature of the measurements will allow monitoring crop growth through time and the development of new dynamic traits from time series analysis that may provide a deeper understanding of phenotypic and genotypic variation for complex traits associated with growth and development.

## Author contributions

JJ-B and DD: Designed and commissioned Phenomobile Lite; JJ-B: Designed the LiDAR processing algorithms with input from RF and XS; PR-L: Implemented the algorithms and processing pipeline with input from JJ-B; AC: Did the experimental design for the field experiments; JJ-B, WB, and GR: Did the statistical analysis; JJ-B and DD: Wrote the manuscript with contributions from GR, RF, XS, WB, and RJ.

### Conflict of interest statement

The authors declare that the research was conducted in the absence of any commercial or financial relationships that could be construed as a potential conflict of interest.
